# Hippocampal inflammasome enrichment in intermediate Alzheimer's disease: Strong spatial findings, but clearer stage and mechanistic definition are needed

**DOI:** 10.1111/bpa.70103

**Published:** 2026-06-07

**Authors:** Chenhong Zeng, Baodong Wang, Yehua Bao

**Affiliations:** ^1^ Department of Acupuncture and Rehabilitation Hangzhou TCM Hospital Affiliated to Zhejiang Chinese Medical University Hangzhou China; ^2^ Department of Gastroenterology The First Affiliated Hospital of Zhejiang Chinese Medical University (Zhejiang Provincial Hospital of Chinese Medicine) Hangzhou China

Dear Editor,

We read with great interest the study by de Rivero Vaccari et al. on inflammasome signaling in selective brain regions during the intermediate stage of Alzheimer's disease (AD) [1]. The authors compare the hippocampus, temporal cortex, and frontal cortex, and combine gene profiling with protein and imaging data. Their central message is clear and important: inflammasome‐related signals are strongest in the hippocampus, are linked to neuritic plaque‐rich areas, with sex‐stratified analyses suggesting a possibly stronger signal in some female subgroups. This work adds useful detail to the growing literature on innate immune activity in AD.

The study has several strong points. The regional design is a major advantage, because AD pathology does not spread evenly across the brain [2]. The finding that the hippocampus shows the largest rise in inflammasome‐related genes and proteins fits well with the known early damage of this region. The use of a broad marker set, including ASC, NLRP1, NLRP3, AIM2, caspase‐1, and GSDMD, also gives the paper a wider scope than many earlier reports. In addition, the use of pTau217 is timely and adds clinical value, since this marker is gaining attention in AD diagnosis [3].

At the same time, several issues deserve closer attention. First, the definition of the intermediate AD group remains broad. The study combines cases across Braak stages III to V [1]. This range likely includes brains at different points of disease spread. Because the main claim of the paper is regional selectivity, stage mixing may weaken the precision of the conclusion. A case at Braak III is not equal to a case near Braak V in terms of cortical tau burden, plaque load, and inflammatory state [4, 5, 6]. A stage‐stratified analysis, even if only exploratory, would help show whether the hippocampal signal is stable across the whole intermediate range or driven mainly by later cases within that group.

Second, the link between inflammasome activation and pyroptotic death should be stated more carefully. The increase in GSDMD staining is interesting, but it does not by itself prove active pyroptosis [7]. Stronger support would require evidence of the cleaved GSDMD fragment, active caspase‐1, and mature IL‐1 family cytokines. Without these data, the findings support a pyroptosis‐related pattern, but not a firm conclusion that pyroptotic cell death is taking place. This point becomes more important because the paper moves from region‐based gene data to a stronger mechanistic claim about cell death.

A related issue is regional matching across assays. The transcriptomic work focuses on the hippocampal CA1 region, whereas the GSDMD imaging analysis is taken from CA2 [1]. Since hippocampal subfields differ in anatomical and molecular organization, and also show differential vulnerability to tau pathology, this shift may affect interpretation [8]. Direct comparison of molecular and imaging results is strongest when the same anatomical field is used across methods. Clarifying why different subfields were chosen, and whether the same pattern is present in both, would strengthen the paper.

Third, the imaging results are suggestive, but the cellular meaning of colocalization still needs sharper definition. The authors show that NLRP3 is mainly seen in microglia, whereas NLRP1 is linked to neuronal processes, and AIM2 appears in plaque‐rich areas with ASC. This is valuable. Still, neuritic plaques are crowded sites where signals from nearby cells can appear to overlap. For that reason, visual overlap alone is not enough to show that these sensors act in the same compartment or even in the same cell. More quantitative colocalization analysis, or higher‐resolution spatial methods, would make the cellular model convincing.

The sex‐based findings are also of interest, but they should be framed as early evidence rather than a settled biological pattern. A stronger female‐associated signal is plausible in some regions, but the overall cohort is modest and sex‐stratified comparisons likely have limited power. This part of the study is best viewed as hypothesis‐generating and should be confirmed in larger series.

Finally, the translational message of the paper is promising but preliminary. Because the study is cross‐sectional and postmortem, it cannot show whether inflammasome signaling starts pathology, speeds it up, or rises as a late response to tissue injury. This limit should be stressed more clearly. Independent replication will also be important before these findings are moved toward biomarker or treatment work.

In summary, this study makes a useful contribution by showing that inflammasome‐related changes in intermediate AD are not uniform across brain regions and are most evident in the hippocampus. It also raises an important question about how plaque pathology, tau injury, and innate immune signaling interact in space. With clearer stage definition, stronger proof of pyroptosis, better anatomical matching across assays, and more precise cell‐level analysis, the authors' model would become more persuasive. We believe this paper will encourage further work on regional immune mechanisms in AD and help shape focused biomarker and treatment studies.

## FUNDING INFORMATION

This work was supported by the Joint TCM Science & Technology Projects of National Demonstration Zones for Comprehensive TCM Reform (GZY‐KJS‐ZJ‐2025‐099).

## CONFLICT OF INTEREST STATEMENT

The authors declare no conflicts of interest.

## Data Availability

Data sharing is not applicable to this article as no datasets were generated or analyzed during the current study.
